# An Assessment of Six Muscle Spindle Models for Predicting Sensory Information during Human Wrist Movements

**DOI:** 10.3389/fncom.2015.00154

**Published:** 2016-01-14

**Authors:** Puja Malik, Nuha Jabakhanji, Kelvin E. Jones

**Affiliations:** ^1^Department of Biomedical Engineering, University of AlbertaEdmonton, AB, Canada; ^2^Faculty of Physical Education and Recreation, University of AlbertaEdmonton, AB, Canada; ^3^Neuroscience and Mental Health Institute, University of AlbertaEdmonton, AB, Canada

**Keywords:** proprioception, Ia afferent, sensorimotor control, spike train, entropy

## Abstract

**Background:** The muscle spindle is an important sensory organ for proprioceptive *information*, yet there have been few attempts to use Shannon information theory to quantify the capacity of human muscle spindles to encode sensory input.

**Methods:** Computer simulations linked kinematics, to biomechanics, to six muscle spindle models that generated predictions of firing rate. The predicted firing rates were compared to firing rates of human muscle spindles recorded during a step-tracking (center-out) task to validate their use. The models were then used to predict firing rates during random movements with statistical properties matched to the ergonomics of human wrist movements. The data were analyzed for entropy and mutual information.

**Results:** Three of the six models produced predictions that approximated the firing rate of human spindles during the step-tracking task. For simulated random movements these models predicted mean rates of 16.0 ± 4.1 imp/s (mean ± *SD*), peak firing rates <50 imp/s and zero firing rate during an average of 25% of the movement. The average entropy of the neural response was 4.1 ± 0.3 bits and is an estimate of the maximum information that could be carried by muscles spindles during ecologically valid movements. The information about tendon displacement preserved in the neural response was 0.10 ± 0.05 bits per symbol; whereas 1.25 ± 0.30 bits per symbol of velocity input were preserved in the neural response of the spindle models.

**Conclusions:** Muscle spindle models, originally based on cat experiments, have predictive value for modeling responses of human muscle spindles with minimal parameter optimization. These models predict more than 10-fold more velocity over length information encoding during ecologically valid movements. These results establish theoretical parameters for developing neuroprostheses for proprioceptive function.

## Background

The realization of restoring movement using brain-machine interfaces has begun and there is a great deal more work needed to refine and develop this technology (Wessberg et al., 2000; Nicolelis, 2003; Donoghue et al., 2004; Schwartz, 2004). An area of current development is the incorporation of sensory feedback from proprioceptors, or artificial proprioceptor-like sensors (Clark et al., 2014; Fisher et al., 2014, 2015; McGee et al., 2014; Niu et al., 2014). One of the afferents involved in proprioception is the primary (Ia) afferent innervating the specialized sensory structures in skeletal muscle: muscle spindles (Gandevia, 1996; Prochazka, 1996). It is our belief that studying the sensory coding schemes that have evolved in muscle spindles will be important for developing biomimetic prosthetics.

Information theory has been a useful tool to estimate the capacity of communication channels in engineering and design codes that take advantage of that capacity (Shannon, 1948). Engineering closed-loop neuroprosthetics with proprioceptive sensory encoding would benefit from measuring the information about movement encoded in the firing rate of the muscle spindle afferents. These data can be used to estimate the bandwidth of proprioceptive feedback in the intact sensorimotor system that is being replaced with a neuroprosthetic device. Information theory also provides an estimate of the precision of the neural code, informing the choice of resolution of synthetic sensors (McGee et al., 2014) or stimulus parameters for activating intact sensory systems (Clark et al., 2014; Fisher et al., 2014). To characterize the potential information content it is important to capture the full entropy of the sensory source that would be encountered in ecological conditions. In the visual system investigations of neural coding moved from synthetic input, e.g., oriented bars and sinusoidal gratings, to natural stimuli improving the decoding algorithms (Simoncelli and Olshausen, 2001; Olshausen and Reinagel, 2003). We propose that the same approach should be used to define the information content of the sensory systems underlying proprioception. Prior to collecting experimental data, our intention was to predict the sensory information encoded in muscle spindle firing during movements of the wrist joint in humans. To make these predictions, a plausible model of human muscle spindle firing in response to movement needed to be determined.

Quantitative models based on cat muscle spindle physiology started in the late 1960s (Matthews and Stein, 1969) and continued slowly over the next three decades (Houk et al., 1981; Hasan, 1983; Scott and Loeb, 1994; Prochazka and Gorassini, 1998b; Mileusnic et al., 2006; Niu et al., 2014). To date, the mathematical models of muscle spindle primary afferents have only been tested with data sets acquired in acute and chronic cat experiments. It is imperative that these models be tested against responses from human muscle spindles, especially given suspected differences between species (Prochazka, 1999). Thus a secondary aim of this study is to determine whether mathematical models based on the cat, have predictive value for human muscle spindle responses. We chose to follow the approach of Prochazka and Gorassini (1998b) who compared the performance of six models to muscle spindle data from cat hamstring muscles during locomotion: a behavioral task with natural sensory statistics. In that study there was only a modest improvement from adding an EMG signal to mimic coactivation of gamma and beta motor neurons. Therefore, we have excluded this parameter in our initial study of these models. The six models were assessed in relation to the ensemble firing rate profile of eight human extensor carpi radialis (ECR) muscle spindes (presumed group Ia) recorded during a step-tracking, center-out task. Our emphasis is on a first order assessment of the utility of these models for capturing basic features of the step-tracking task and then using the models to predict the response during a more complex task: random two-dimensional tracking (Paninski et al., 2004).

## Methods

### Overview

**Figure 1:**
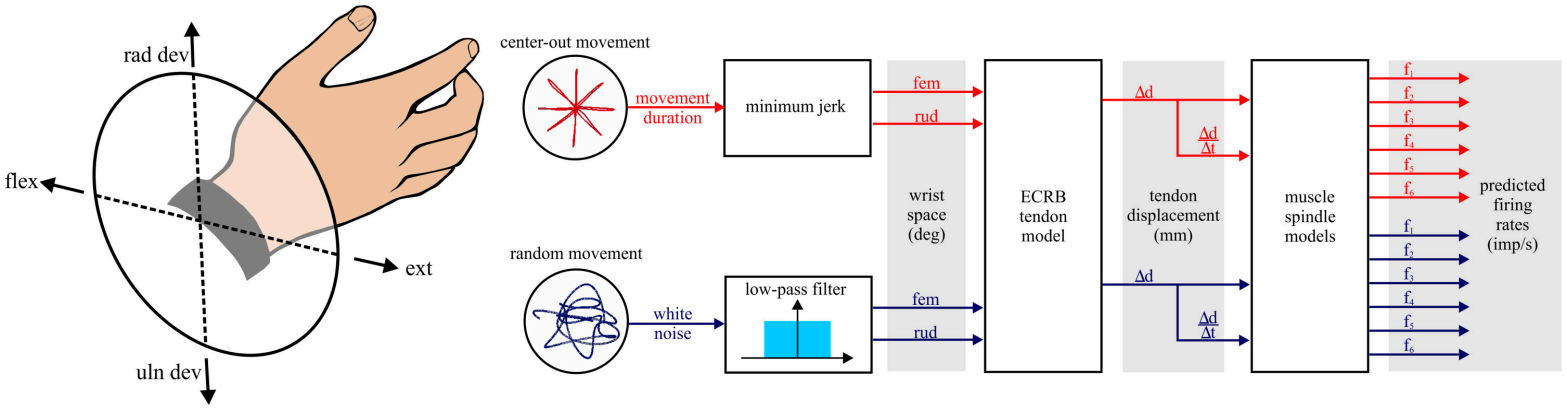
**Schematic of the steps involved in the simulations**. All simulations are hypothetical movements of the wrist in right-hand coordinates. The wrist joint coordinate system is illustrated to orient the reader to subsequent polar plot figures where angle is determined by the rotation of the wrist about its two axes. Two types of task are examined: center-out and random movements. fem, flexion/extension movement; rud, radial/ulnar deviation movement; ECRB, extensor carpi radialis brevis muscle.

The simulations and models developed for this study are schematically illustrated in Figure 1. The first step was generation of hypothetical wrist movements for two different tasks: center-out and random movements. The kinematics of the wrist movements were simplified to rotations about two orthogonal and intersecting axes. The movements were measured in degrees with respect to a neutral position defined as the origin of a co-ordinate system with flexion/extension movements along the x-axis (extension being positive) and radial/ulnar deviation movements along the y-axis (radial deviation being positive). This Cartesian system was transformed into a polar co-ordinate system. In the polar system pure extension movements pointed toward the right at 0°, pure flexion movements to the left at 180°, radial deviation movements pointed upwards at 90°, and ulnar deviation movements pointed downwards at 270° (Figure 1, left).

The resulting rotations of the wrist were input to a model of tendon displacement for the extensor carpi radialis brevis (ECRb) muscle (Loren et al., 1996). The tendon displacement output was then used as input to six different muscle spindle models to generate predicted Ia afferent firing rate. These models have been evaluated in relation to the natural movements during cat locomotion and exhibited good fits to firing rate profiles of ensembles of cat Ia afferents (Prochazka and Gorassini, 1998a,b). For our goal of predicting sensory feedback from Ia afferents during natural human wrist movements, these models seemed a clear choice. A more detailed and comprehensive model was not included in our assessment (Mileusnic et al., 2006). A major goal of this model is the accurate modeling of fusimotor effects, which we did not investigate in this initial assessment. In addition, the length input to this model is fascicle length (in units of optimal muscle fascicle length). Converting from our approximation of tendon displacement to fascicle length was dubious given the sparse experimental data from the human ECR muscle.

All simulations and analysis were performed in Matlab ver 7.0.4 and Simulink ver 6.2 with a time step of 1 ms.

### Center-out movement simulations

The center-out task is a staple of sensorimotor neurophysiology and has been used extensively for 2D and 3D reaching experiments in monkeys and humans (Georgopoulos et al., 1982; Gordon et al., 1994). This task, also referred to as step-tracking, has been intensively studied during wrist movements by Hoffman and Strick in both man and monkey (Hoffman and Strick, 1986a,b, 1990, 1993, 1999; Kakei et al., 1999). Our simulations of afferent responses during the center-out task build on the wide-spread use of these movements for studying sensorimotor systems.

The kinematics of the center-out movements were calculated to eight equally spaced targets around a circle in wrist joint space (Figure 1). Minimum jerk trajectories were used as they are descriptive of wrist movements (Stein et al., 1988) and similar to the kinematics predicted by a more accurate causative optimal control model for the wrist (Haruno and Wolpert, 2005). The amplitude of the movements was 7° of joint rotation to match with a subset of data extracted from a previous human muscle spindle study (Jones et al., 2001). The simulations included a short period of 500 ms in the neutral wrist position followed by a movement phase of 650 ms that followed the minimum jerk trajectory (Flash and Hogan, 1985). The simulations continued for a period of 500 ms after reaching the target. The average velocity of movement to the targets was 10.8 deg/s with a peak velocity equal to 20.7 deg/s. These movement velocities correspond to those previously reported for the human data: average 9–10 deg/s with peak velocities of 20–30 deg/s (Jones et al., 2001).

### Center-out movement—human data

Eight ECR muscle spindle afferents were selected from a larger sample of data to compare with the predicted firing rate of the spindle model. This subset of data were selected because: the data were from putative Ia afferents in the ECR muscle; the responses were obtained during a center-out task with kinematics that approximate minimum jerk trajectories; and responses were obtained during movements to all eight targets. In a previous study of the ensemble response of spindle primary afferents in the cat hamstring, the authors reported that after five or six had been averaged to estimate a population response, the addition of more units did not change the population response significantly (Prochazka and Gorassini, 1998b). On this precedent, we felt confident that our data sample was sufficient to estimate general ensemble firing rate behavior of human ECR spindle primary afferents. The spike trains were aligned to movement onset and the average movement duration was 634 ± 19 ms (mean ± *SD*). To calculate the mean firing rate and 95% confidence intervals for the ensemble of ECR afferents, the individual single-trial spike trains were convolved with a Gaussian kernel then averaged. The width of the kernel function (60 ms) was chosen iteratively considering the phasic response and background discharge rate while comparing the ensemble to the predicted firing rates from the six muscle spindle models.

### Random movement simulations

While the center-out task has been widely used in sensorimotor neurophysiology to investigate neural coding, there are a number of shortcomings that have been noted (Paninski et al., 2004). First, these movements, as with all point-to-point movements, are relatively straight and exhibit bell-shaped velocity profiles where the peak velocity is proportional to the target distance. These invariant features of movement result in the coupling of position and velocity, two variables that can have independent effects on responses of muscle spindles and proprioception. Another difficulty with the center-out task is that the data are statistically non-stationary, i.e., the mean and variance change over time during the task. The non-stationarity precludes the use of any analysis methods that require a stationary stochastic time series; information-theoretic analysis for example. In addition to the inevitable coupling of position and velocity and the non-stationary data, the center-out task results in movements that occupy a limited amount of the available joint space, and while natural, do not approximate the statistics of ergonomic movements of the wrist. For these reasons, we decided to simulate a random pursuit-tracking task introduced to sensorimotor neural coding studies (Paninski et al., 1999, 2004).

The goal of the random movement simulations was to reproduce the statistics of human wrist movements during typing (Serina et al., 1999). The random movements were generated by a Gaussian random number generator so that the standard deviation of position matched the ergonomic data (Table 1). In order to match the statistics of wrist velocity during typing, a 6th-order low-pass Butterworth filter with cut-off frequency of 1.5 Hz was applied.

**Table 1:** **Wrist movement statistics**.

	**Position**		**Velocity**	
	**Typist data (deg)**	**Simulations (deg)**	**Typist data (deg/s)**	**Simulations (deg/s)**
Flexion/Extension	19.9 ± 6.0	0 ± 6.0	25 ± 12	7.1 – 21.1
Radial/Ulnar deviation	18.6 ± 4.1	0 ± 4.1	11 ± 7	5.1 – 14.4

*Typist data was given as mean ± within-subject SD (Tables 2, 3.) and the probability distribution of position was assumed to be Gaussian. The non-zero mean joint position was not a parameter for the spindle models so it was zeroed in the simulations and the standard deviations were matched. Velocity was rectified prior to calculating statistics: mean ± SD for data; range of median velocities for simulations*.

**Figure 2:**
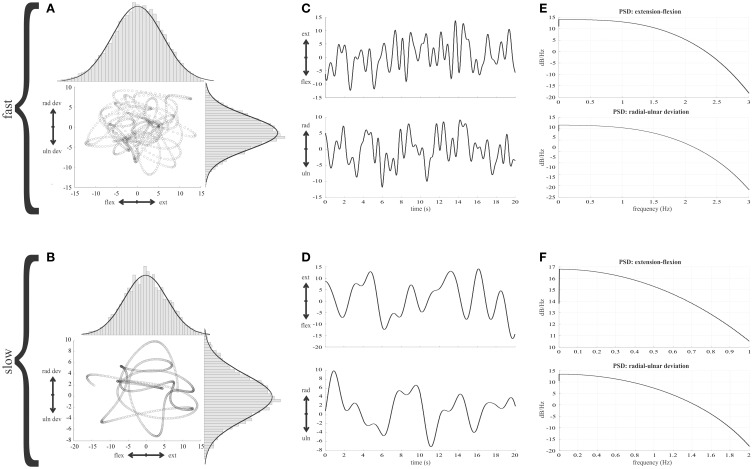
**Features of the random wrist movement simulations. (A,B)** The trajectory of the random wrist movements in 2D wrist joint space is illustrated for the fastest (1.5 Hz cut-off) and slowest (0.5 Hz cut-off) movement speeds. The histograms illustrate the distribution of angles about the two wrist axis and a Gaussian curve is overlaid to illustrate the distribution of position is the same at the two different speeds. **(C,D)** Thirty seconds of the wrist position time series for each wrist axis to illustrate the random nature of the simulated movements. **(E,F)** Power spectral densities of wrist position in both wrist dimensions for the 1.5 and 0.5 Hz signals respectively.

Given the importance of the simulations of the random movements, we have illustrated the key features in Figure 2. Gaussian curves overlying the histograms (Figures 2A,B) illustrate that a constant position distribution was conserved in the final random wrist movement signals. The curves have the same parameters for a given axis at fast and slow speeds. Power spectral analysis was carried out for position and velocity along each wrist axes to check for a flat spectrum below eleven cut-off frequencies: 0.5–1.5 Hz in 0.1 Hz increments. The flat nature of the power spectral density plots indicated the signals were approximately white within the band-pass of interest and therefore considered random (Figures 2E,F).

### Tendon displacement model

The rotations about the two axes of the wrist were used to calculate the tendon displacement of the ECRb muscle resulting from the movements. These calculations were based on a human cadaveric forearm study that reported equations for instantaneous moment arms with respect to joint rotation (Loren et al., 1996). The equation for ECRb, in the neutral wrist posture, was integrated to generate an equation for instantaneous tendon displacement (in mm) with respect to joint rotation. Tendon displacement and velocity, computed with the transfer function 500 s/(s+500) where s is the Laplace operator, were then used as input to the six muscle spindle models (Figure 1). Corrections for compliance or muscle fiber pennation were not features of the spindle models tested, so were ignored for the present study.

**Figure 3:**
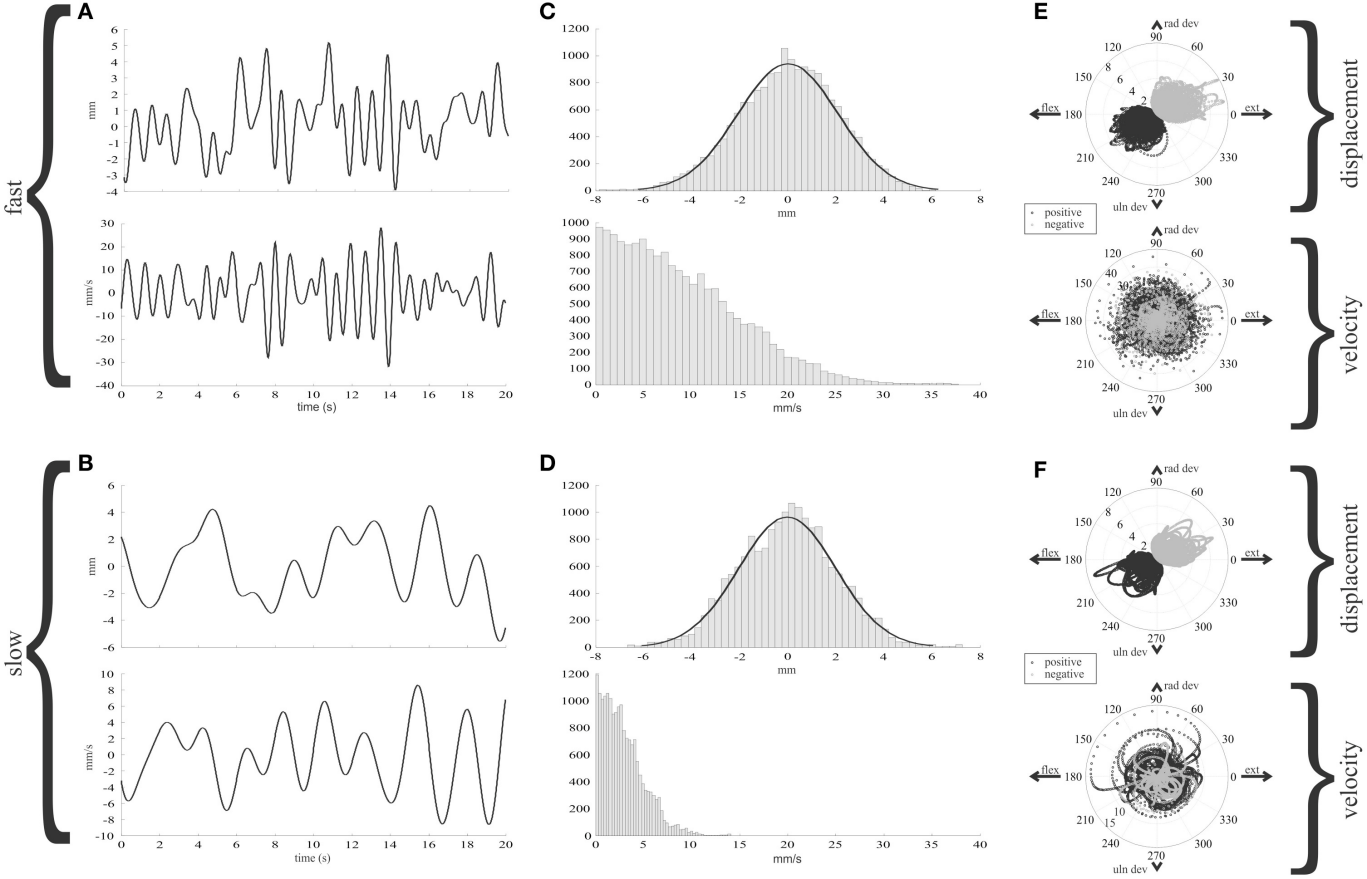
**Features of ECRb tendon displacement and velocity during random human wrist movements**. The simulated random movements result in Gaussian distributed inputs to the muscle spindle models. The distribution of tendon displacement is constant across the slow to fast movement speeds, whereas the velocity increases. Since the simulated movements are constrained to the statistics gleaned from ergonomic studies of wrist movements, these stimuli can be considered to have a statistically natural distribution. **(A,B)** tendon displacement (above) and velocity (below), for fast (1.5 Hz cut-off) and slow (0.5 Hz cut-off) random wrist movements. **(C,D)** Histograms of tendon displacement and rectified velocity. At the faster movement, the tendon velocity distribution spreads out to higher values. **(E,F)** Polar plots of tendon displacement and rectified velocity (below) for 1.5 Hz **(E)** and 0.5 Hz **(F)**. Positive displacement or velocity is black, negative is gray.

The tendon displacement and velocity during the random wrist movements are illustrated in Figure 3 for the fastest and slowest speeds. The key feature of this figure is that it illustrates the range and distribution of the two inputs to muscle spindles during statistically natural human wrist movements. These data allow comparison of the statistics of tendon movements in humans and that in other species, e.g., cats during locomotion. In the middle column the velocity distribution has been rectified prior to calculating the median value at each of the 11 different filter rates. A Kurskal-Wallis test showed a significant difference in the median tendon velocities as a function of filter rate (*p* < 0.05). The last column illustrates polar plots where the angle is determined by the position in wrist joint space and the distance to a point is the value of tendon displacement or velocity. The polar plots of displacement illustrate the ECRb tendon is stretched (positive displacement, black) for wrist positions combining flexion and ulnar deviation and shortened for wrist positions in the opposite direction. Tendon velocity is independent of wrist position and can have positive or negative values anywhere in wrist joint space. This illustrates a *key objective* of the random movement simulations, uncoupling of wrist joint position, and tendon velocity that are unavoidably linked in the center-out task.

### Predicted Ia afferent firing rates

Six muscle spindle models were used to generate predicted firing rate time series resulting from the simulated movements (Matthews and Stein, 1969; Chen and Poppele, 1978; Houk et al., 1981; Hasan, 1983; Prochazka and Gorassini, 1998b). All of these models were shown to have some predictive value for estimating ensemble Ia afferent firing rate during normal cat locomotion, though when compared with slow ramp-&-hold stretches one emerged as the most general and accurate. The muscle spindle models were implemented in Simulink, using a previous published block-diagram (Figure 7 in Prochazka and Gorassini, 1998b). As the models were no longer available from the cited internet site, correct implementation was verified by comparing model behavior to the published responses during cat locomotion (Figure 3 in Prochazka and Gorassini, 1998b). The solver used in our simulations was the Euler algorithm with a step-size of 1 ms. For comparison to the human data set, the baseline firing rate of the models was set to 10 imp/s from its original 82 imp/s. This decision was made after averaging the mean firing rate for each of the eight human muscle spindles while holding in the central starting position: 10.2 (*SD* 1.4) imp/s. Our goal was to make the *minimal* adjustment to the models rather than leaving parameters free and running a traditional optimization algorithm. This was done to evaluate if the models had predictive value with minimal change, to test the hypothesis that there is little difference between cat and human muscle spindle responses.

### Data analysis

#### Center-out

The most wide spread approach to analysis of neurophysiological data during the center-out, or step-tracking, task is the “mean vector” analysis method by which the directional tuning is determined (Georgopoulos et al., 1982; Gribble and Scott, 2002). This approach has been extensively used for analysis of muscle spindle coding during passive movements of the ankle (Bergenheim et al., 2000; Roll et al., 2000, 2004; Ribot-Ciscar et al., 2002, 2003) as well as our previous study of active and passive movements of the wrist (Jones et al., 2001). The length of the mean vector was normalized to values between 0 and 1. To test for significance of directional tuning, a bootstrap test was used where the mean rate and target angle are resampled from the original data (4000 resampling trials) and the resulting resampled vector length is compared to the original. If fewer than 200 resampled vectors are longer than the original, the directional tuning is considered significant with 95% confidence. Note, resampling is not done if the original mean vector length is <0.001, as this already indicates a non-directional distribution in circular statistics (Batschelet, 1981).

#### Quantitative comparison of human data and model

Both qualitative and quantitative comparisons were done to evaluate the ability of the six muscle spindle models to capture the dynamics of the human experimental data during the center-out task. Root mean square (RMS) error was calculated between each model and the ensemble data on the following measures: mean firing rate, directional tuning vector length, static index, dynamic index, and temporal similarity. For the first two measures, an RMS error was calculated during two phases of the task: (1) movement to the target, and (2) holding on the target. The RMS error for a particular measure was normalized by dividing by the median RMS error for the six models. The normalized errors were summed over all the measures to give a total error score. The error score has a lower limit of zero, which corresponds to perfect prediction, and an unbounded upper limit. The consolidated error score was used to rank order the six models in their ability to predict the human ensemble data. Most of these measures are self-explanatory with full details given in Malik (2006). The measure of temporal similarity evaluated the difference between the ensemble instantaneous firing rates and the firing rates predicted by the models over intervals of 100 ms. The squared difference in firing rate at each interval was summed over the duration of a trial to each of the eight targets. The final RMS error for each model was computed by dividing the sum of squared differences by the number of 100 ms bins and number of targets, then taking the square root.

#### Random movements

The random movement data was analyzed using entropy and mutual information for a continuous channel (Shannon, 1948). This approach to analysis of sensory stimuli in neural systems has proven to be a powerful framework for nonparametric and nonlinear analysis (e.g., Bialek et al., 1991; Bialek and Rieke, 1992). Following Shannon we adopt the logic that information = uncertainty = entropy and, using base 2 for our logarithms, interpret the entropy as the smallest number of bits needed to communicate our signal of interest, which is tendon displacement or velocity. The ability to transmit these signals that convey the mechanical state of the muscle using muscle spindle firing rate was assessed by mutual information. This quantity measures the average number of bits an observer receives about the tendon displacement or velocity by observing firing rate. The probability density function for a signal was estimated from a histogram of the signal after which entropy was calculated. Mutual information is a linear addition of the respective entropies, *I*_*xy*_ = *H*_*x*_ + *H*_*xy*_, where H is entropy, x is the input signal (tendon displacement or velocity) and y is the firing rate of one of the six muscle spindle models. The relevant equations can be found in Shannon (1948), and have been reproduced in other sources (e.g., Cover and Thomas, 1991; Rieke et al., 1999). Calculations were done in Matlab using (Moddemeijer, 1989).

## Results

### Center-out movements

In this section we assess whether a group of six muscle spindle models, which have been developed and used to interpret data from cat studies, give a general and accurate prediction of the responses of human muscle spindles during a step-tracking task.

#### Comparing time series data

**Figure 4:**
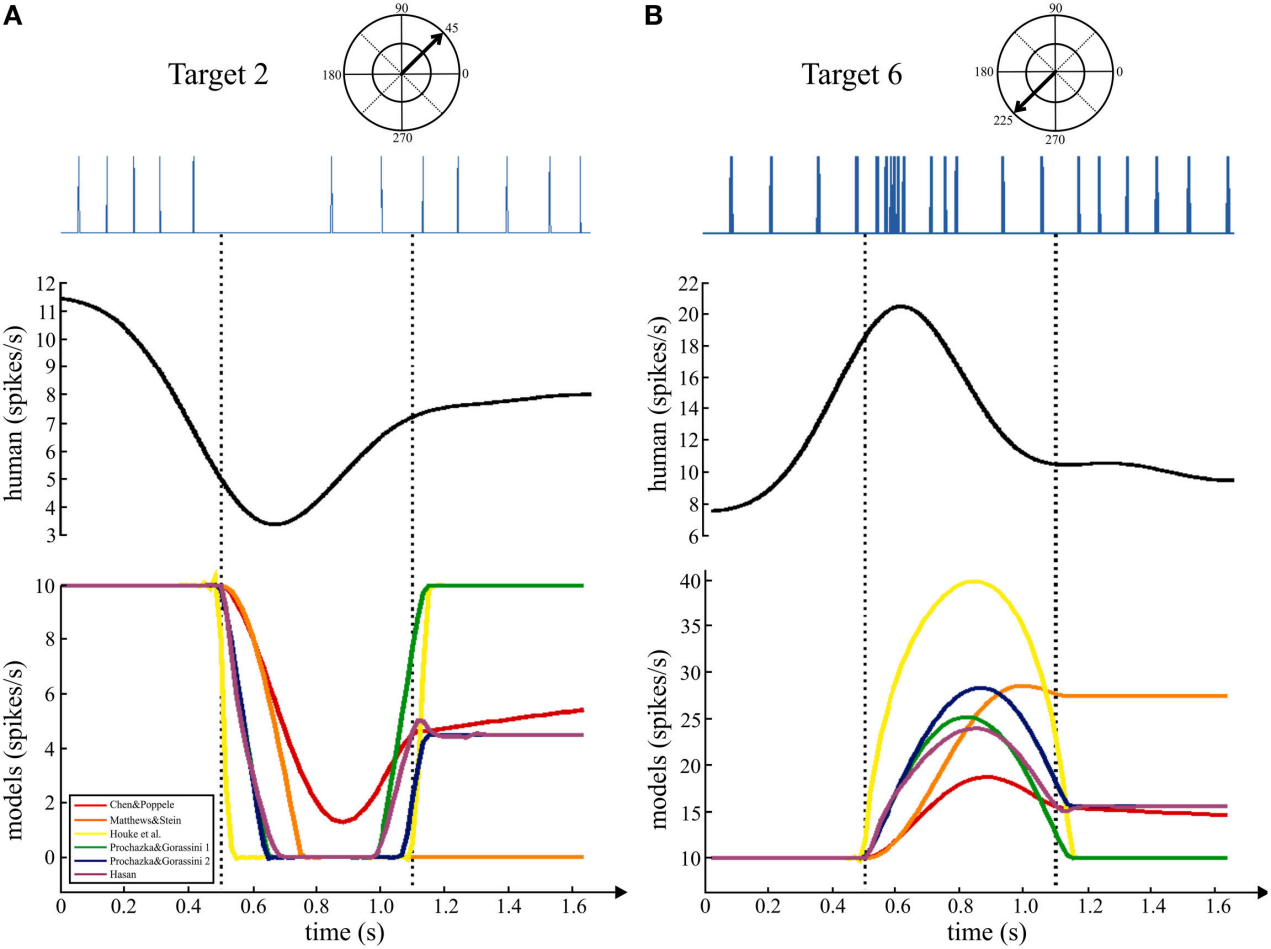
**Comparing movements to opposite targets**. Upper trace shows an example spike train from a human ECR muscle spindle during movements to two targets. Middle trace shows the continuous firing rate generated from the ensemble data of 8 ECR spindle afferents. The first dashed line indicates the onset of movement to the targets and the second is 650 ms later, the end of the minimum jerk movement phase.

In the human data set that was analyzed for this study, the subjects were asked to move quickly to the targets with an emphasis on accuracy. The main movement phase to the target was in a straight line with a bell-shaped velocity. Movements to some targets, for which the ECR muscle is the primary agonist, resulted in a decrease in firing rate of the human muscle spindles as illustrated in Figure 4. During movement to target 2 (negative tendon displacement, Figure 3E), this muscle spindle was temporarily unloaded resulting in a pause in the spike train (Figure 4A, left upper trace). A continuous estimate of firing rate generated from the ensemble firing rates of all eight spindles in the data set showed a transient period of decreased firing rate during movement followed by recovery of firing rate during the static hold-target phase (Figure 4A, left middle trace). Movement in the opposite direction to target 6 (positive tendon displacement, Figure 3E) resulted in a burst of action potentials from the muscle spindle afferent with instantaneous firing rates reaching almost 90 imp/s followed by adaptation to a lower rate during the static phase. The continuous firing rate estimate from the ensemble showed a clear separation of dynamic and static firing rate responses during “movement to” and subsequent “holding on” the target.

The changes in firing rate predicted by the models during movements to these same targets had similar dynamic and static phases. During movements to target 2, five of the six models showed a transient decrease in firing rate during movement with recovery of firing rate during the static phase of holding on the target. Simulations of movements to target 6 showed a transient increase in predicted firing rate that was higher than the rate after reaching the target. The firing rates predicted by four of the models during the dynamic phase were of similar magnitude to the human data, but only three of these predicted rates similar to the human example in the static phase.

**Figure 5:**
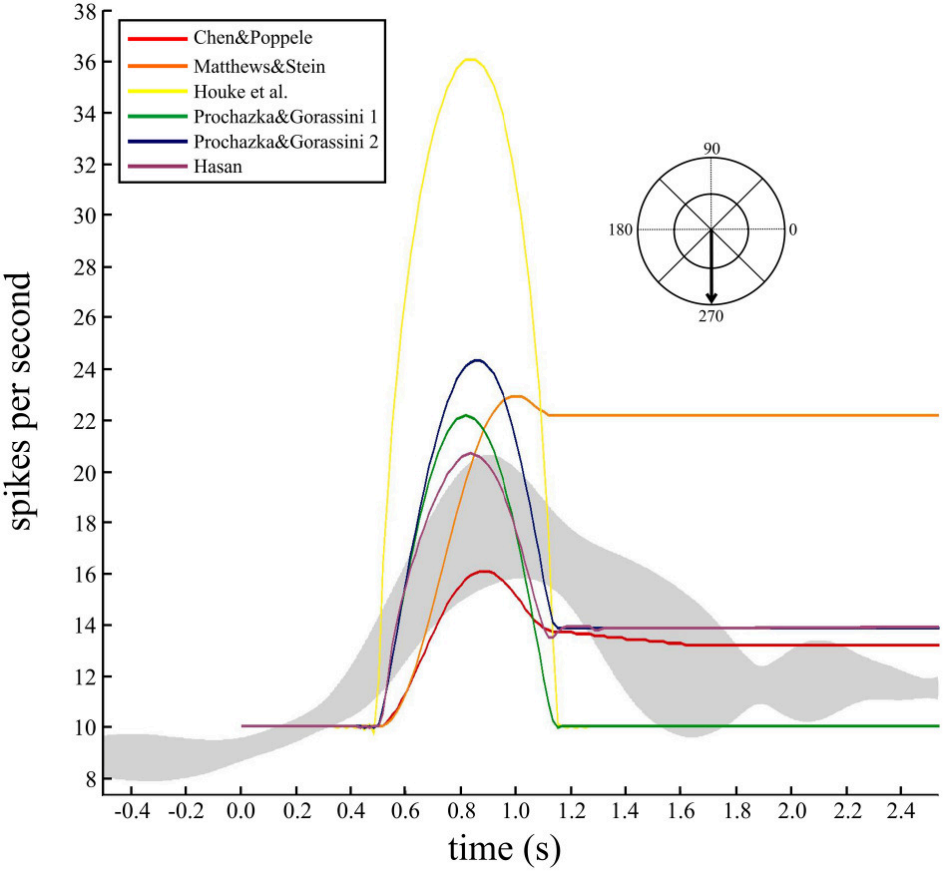
**Comparing the temporal dynamics of firing rate and variability for a human muscle spindle and six models**. Continuous firing rate responses from a single human ECR spindle, recorded during four repeat movements to the target at 270°. A smoothed response was generated from the spike trains using a Gaussian kernel (60 ms bandwidth) and the five repeated movements were averaged to find the mean response and 95% confidence intervals (gray band). The movement duration was 662 ± 95 ms (mean, *SD*). Overlaid are predicted firing rates from the six models for a minimum jerk trajectory (650 ms duration) to the same target. Baseline firing rate in the models was set to 10 imp/s and the average for the human data was 8.5 imp/s. Records are aligned at movement onset (0.5 s).

While the individual movements to these two targets were nearly 100 ms shorter than the simulated movements (650 ms), the grouped data for all eight spindles during movements to all targets was 634 ± 19 ms (mean ± *SD*). Therefore, the durations in the experimental data and simulations are of a similar magnitude to allow qualitative comparison. To check how well the models predicted the temporal behavior and variability during this task, the responses of a single spindle during four repeated movements to the same target were averaged (Figure 5). The data were aligned to movement onset and the average firing rate with 95% confidence intervals calculated (Figure 5, gray band). The human spindle showed a clear dynamic phase where the average maximum was 18 imp/s, up from a baseline of 8.5 imp/s, and a static component where average rate was 12 imp/s.

Since these movements are well described by a minimum jerk trajectory, we calculated the minimum jerk trajectory to the same target, with movement duration equal to 650 ms (Figure 5). Three of the models showed qualitative features similar to the human data (Figure 5). The remaining three models did not make good predictions of the static phase activity: one was too strong (orange) while the others were too weak (yellow and green) compared to the human data.

These results suggest that some of the models do a better job at predicting the temporal profile of human muscle spindle firing rates during this task. While we have not attempted to match the human kinematics in detail, the predicted minimum jerk trajectories result in predicted firing rates that have clear dynamic and static phases that are similar in magnitude to the average human data. Next we examine whether the models can predict firing rates during movements to all targets in the center-out task.

#### Comparing directional tuning

The human ECR spindle data were analyzed to measure the mean firing rate during the dynamic and static phases of movement to each of the eight targets. Six of the eight afferents were directionally tuned during the dynamic (move) phase (bootstrap, *p* < 0.05) while only three were significantly tuned during the static (hold) phase. The data were then used to estimate the directional tuning for the population. The mean vector for the dynamic phase had a normalized length of 0.28 and an angle of 239°. The mean vector for the static phase was 0.11 long with an angle of 240°. A bootstrap test showed that both vectors were significantly tuned (*p* < 0.05). This result differs from the previous study in which these units were part of a larger sample of ECR spindle afferents. In the previous study, the data were not significantly tuned during the static phase (Jones et al., 2001).

**Figure 6:**
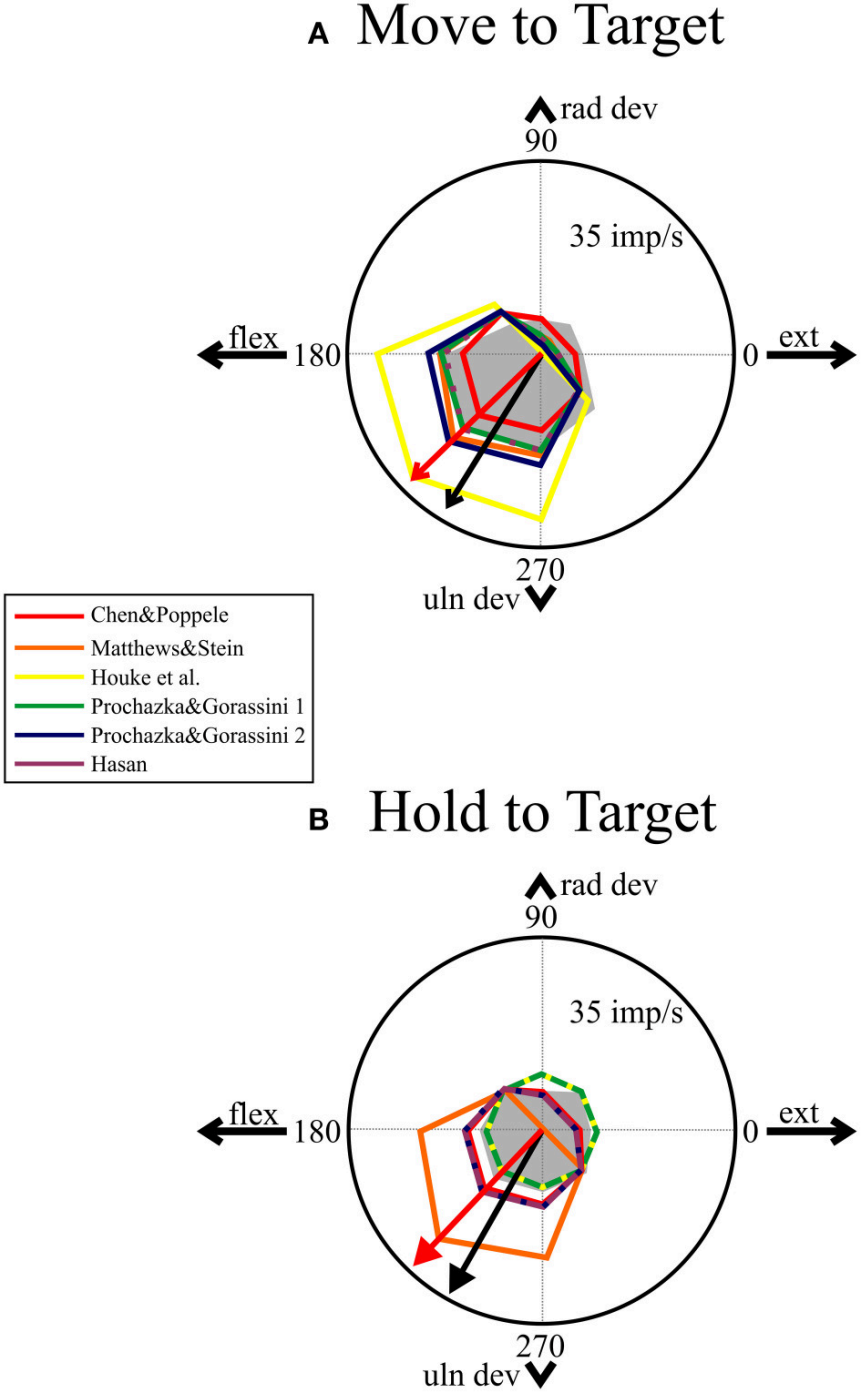
**Comparing directional tuning in polar plots**. The top panel **(A)** shows mean firing rate for the models (see legend) and averaged human data (gray area). The preferred direction of the models (computed by mean vector) was 225° (red arrow) and the preferred direction of the human data was 239° (black arrow). The length of the normalized mean vectors (not illustrated) were: 0.28 (human), 0.28 (red), 0.46 (orange), 0.62 (yellow), 0.45 (green), 0.52 (blue), and 0.46 (purple). The bottom panel **(B)** shows mean firing rates during the static phase of holding on the target. Two models (yellow and green) were not significantly tuned during the hold phase (mean vector length = 0.0), while the others had the same preferred direction (225°) and normalized mean vector lengths of: 0.25 (red), 0.64 (orange), 0.28 (blue), and 0.28 (purple). The mean vector for the human data had an angle of 240° and a length of 0.11, which was significant (*p* < 0.05).

All the models showed directional tuning during the dynamic phase with a mean vector angle of 225°. The lengths of the mean vectors are given in the legend for Figure 6 where the directional tuning is illustrated in polar coordinates. The central gray area illustrates the distribution of the human data and the black arrow the direction of the mean vector. The accuracy of the mean vector predicted by the models (Figure 6 red arrow) was surprising given the variability in the human data and the simplifying assumptions for the biomechanical wrist model and simulations. The human data shows higher firing rates than those predicted by the models in the direction opposite the preferred direction, which could be due to co-activation of gamma or beta motor neurons not accounted for in these simulations.

The data in Figures 4–6 provide qualitative evaluation of the ability of the models to match the human ensemble data. RMS errors were calculated for seven different measures during the center-out task during movements to, and holding on, the eight targets to provide a quantitative comparison of the models. The RMS errors were normalized and summed to produce a total error score, which is a relative measure of the models against each other. These data are presented in Table 2 and indicate that the three models with the lowest error scores in rank order are: red, Chen and Poppele (1978); purple, Hasan (1983); and blue, Prochazka and Gorassini 2 (Prochazka and Gorassini, 1998b). Therefore, these models provide the best overall prediction of the ensemble human muscle spindle data.

**Table 2:** **Overall error for model ranking**.

	**Mean rate**		**Directional tuning**		**Dynamic index**	**Static index**	**Temporal similarity**	**TOTAL error**
	**Move**	**Hold**	**Move**	**Hold**				
C and P (red)	1.0	0.5	0.8	0.5	0.5	0.7	0.8	4.8
M and S (orange)	1.1	2.9	1.6	4.8	0.9	3.5	1.8	16.6
Houk (yellow)	2.1	1.3	2.4	0.1	2.8	2.9	2.1	13.6
P and G1 (green)	0.9	3.4	0.1	0.9	1.4	1.2	0.9	8.7
P and G2 (blue)	1.0	0.7	1.2	1.1	1.1	0.8	1.1	7.0
Hasan (purple)	0.9	0.7	0.5	1.1	0.8	0.8	0.9	5.8

*RMS errors for the seven different measures were calculated then normalized by dividing by the median error of the six models in each column. Errors less than one indicate the model is in the top three for predictive ability for that measure. The last column is the sum of the normalized errors. The red model Chen and Poppele (1978) has the lowest summed error*.

### Random movements

In the previous section we found that previous models of cat muscle spindles could approximate firing rate and directional tuning of a sample of human ECR muscle spindles. The three models with the lowest overall error scores were used to estimate how much information muscle spindle firing rate carries about movement. This was achieved by simulating random movements that dissociate joint position and velocity to maximize the entropy of the stimulus while remaining an ecologically valid input.

#### Prediction of the firing rate time series

**Figure 7:**
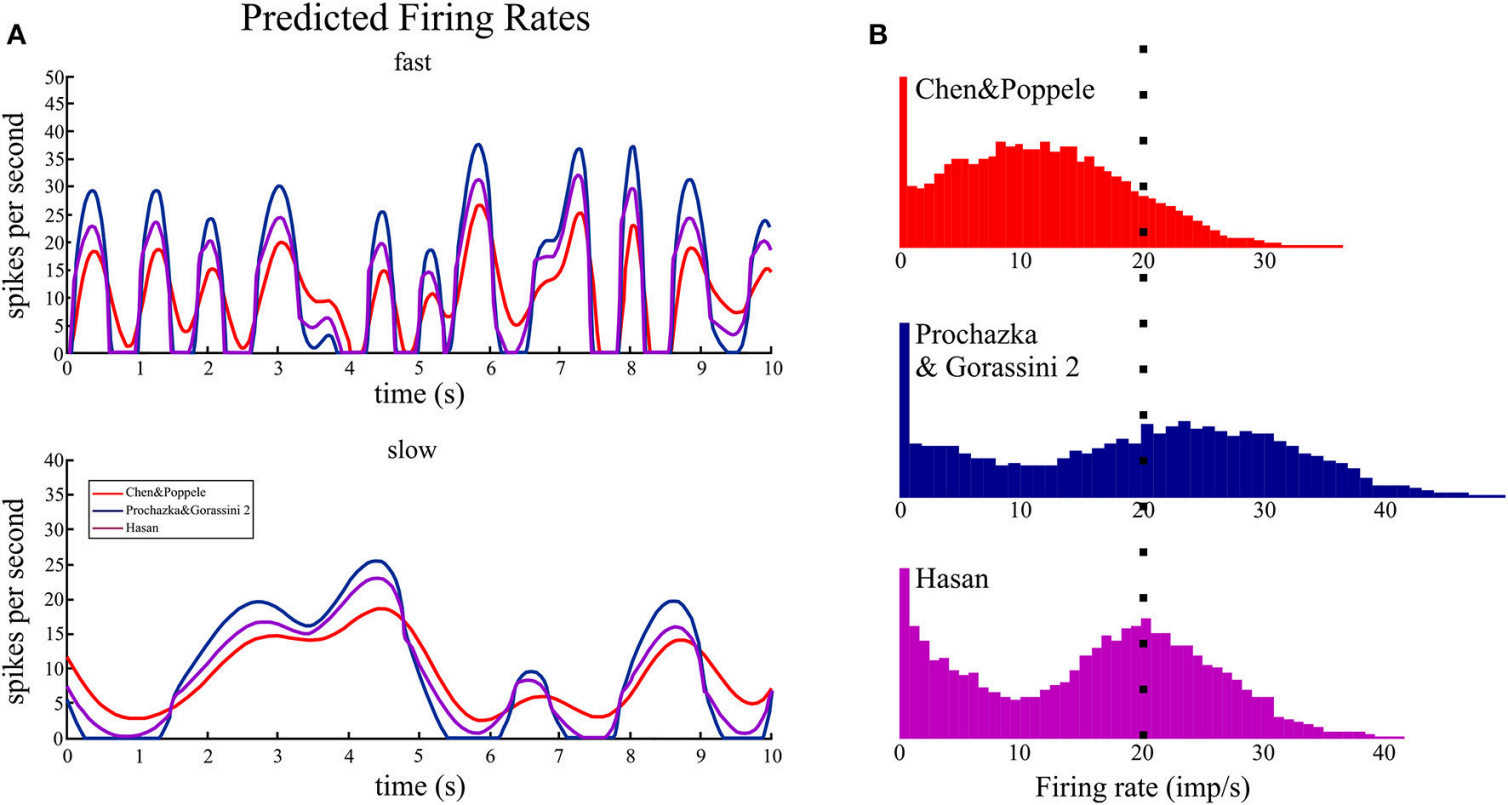
**The time series and histograms of firing rates predicted for random movements**. The random 2D movements in wrist joint space resulted in changes in ECRb tendon length that were input to the muscle spindle models to predict firing rates during movements of the human wrist encountered in normal movements. **(A)**. During fast movements, the predicted firing rates are <40 imp/s. The main distinguishing feature when comparing the three models is the peak rate and periods of silence (i.e., firing rate of zero). Even during slow movements the Prochazka and Gorassini 2 model predicts periods of zero firing rate. **(B)**. The histograms all have the same scale starting at zero firing rate and the dotted line indicates 20 imp/s. The means of these distributions, with and without using zero firing rate in the calculations, are given in Table 3. The shapes of firing rate histograms predicted by the three models are different: unimodal and bimodal. Experimental data during a similar task should be plotted in a similar fashion to evaluate the model predictions.

The time series plots of firing rate predicted by the three spindle models during simulated random movements at two speeds are shown in Figure 7A. The predicted firing rates during the fast movements had a striking “peaky” structure that showed sharp rises to a peak firing rate followed by periods of low to zero firing rates. The primary differences predicted by the three models are the dynamic gain and frequency response with the Chen and Popele (red) model having the lowest dynamic gain and frequency response. The mean predicted firing rates, over 5 min of simulated movement, for the three models ranged from 10.5 to 13.1 imp/s with peak firing rates <50 imp/s (Table 3). Using the mean firing rate to characterize the different models was complicated by periods of zero firing rate. The models predicted periods of unloading, or silencing, that ranged between 12.5 and 35.0% of movement duration. This had a significant effect on the probability distributions for the firing rate signals (Figure 7B). The probability distribution of firing for the Chen and Popele model is unimodal (if the peak at zero is ignored) while the other two models predict a bimodal distribution of firing rates. The differences in distribution are associated with differences in the dynamic gain and frequency response illustrated in the time series plots.

**Table 3:** **Descriptive statistics of firing rates during fast random movements**.

	**C and P (red)**	**P and G 2 (blue)**	**Hasan (purple)**
Mean	10.5 (12.0)	13.1 (20.2)	11.4 (15.9)
Peak	36.4	49.7	41.4
%Silent	12.5	35.0	28.4
H_rate_	4.41	3.85	3.91
I_rate, disp_	0.16	0.08	0.07
I_rate, vel_	0.92	1.33	1.50

*The units for mean and peak firing rates are (imp/s). Mean firing rates were calculated in two ways, with and without (in parentheses) the time during which predicted firing rates were zero. %Silent is the percentage of time during the random movements that firing rate is zero. H_rate_ = total entropy of firing rates (bits). I_rate, disp_ = mutual information between firing rate and tendon displacement. I_rate, vel_ = mutual information between firing rate and tendon velocity. Units for mutual information are bits per symbol. The standard error for mutual information estimates was ≤0.01 in all cases*.

#### Assessing the representation of tendon displacement and velocity using entropy and mutual information

The final goal of this investigation was to characterize the muscle spindle responses to sensory stimuli using information-theoretic analysis. The amount of information in the sensory stimuli was quantified by the entropy of tendon displacement and velocity at each of the eleven random movement speeds. The average entropy and standard error for tendon displacement was 3.05 ± 0.01 bits per symbol across all eleven speeds while the entropy of the tendon velocity signal increased linearly from 3.88 ± 0.01 at the slowest speed to 5.49 ± 0.01 bits per symbol at the fastest speed.

The muscle spindle is a physiological transducer and the mathematical representation of transduction states that the entropy of the firing rates should be less than or equal to the entropy of the source they are encoding (*Theorem 7* in Shannon, 1948). The entropies of the firing rates predicted by the models at the fastest speed (Table 3) are all greater than the entropy for tendon displacement and less than the entropy for the tendon velocity signal. Theoretically, if the models were perfect inverse transducers of displacement and velocity then the entropy of the firing rates would be equal to the sum of their source entropies, in this case 3.05+5.49 = 8.54. Instead the firing rate entropies are about half this value. But these measures of firing rate entropy do not quantify how much information firing rates encode about the stimulus.

To measure the transmission of tendon displacement or velocity information in the firing rates of the models we calculated Shannon's mutual information. The mutual information values were very low between firing rate and tendon displacement (I_rate, disp_ in Table 3). On average about 3.3% of the information about tendon displacement is preserved in the firing rates of the models. In comparison about 22.8% of the information about tendon velocity is preserved in the firing rates of the models during the fastest movements (I_rate, vel_ in Table 3).

## Discussion

Muscle spindles are an important source of sensory feedback that signal information about the position and movement of joints (Stein et al., 2004). Over the past three decades recordings have been made from single muscle spindle afferents in behaving cats, monkeys and humans. The experiments are technically difficult so valid models are helpful to consolidate understanding and guide further experiments. Until now, none of the six models compared here had been tested against a human data set. Our goal in this report was to compare six models against human data and then predict responses expected from human muscle spindles during a novel experimental paradigm: random pursuit tracking.

In simulations of center-out movements, we found that the ensemble firing rate and directional tuning of human ECR Ia afferents was adequately captured by three of the six models: Chen and Poppele (red), Prochazka and Gorassini 2 (P&G2, blue) and Hasan (purple). The three remaining models were considered a poorer representation of the ensemble given the error scores (Table 2).

### Limitations

We have used minimum jerk trajectories rather than fitting to the actual kinematics; we have excluded any terms that would capture phasic gamma or beta motor neuron activity; we have excluded the finer details of the muscle geometry and biomechanics by using tendon displacement as the main stimulus input to the models; and, we have used models that output firing rate as a continuous function rather than a series of action potentials. Despite these simplifications, as a first order approximation these models have clear predictive value for estimating human ECR muscle spindle response during wrist movements. One of the differences we noted between the models and human data was the prediction of zero firing rates during movements opposite to the preferred direction. The human spindle response during movement to target 2 (Figure 4A) was atypical for the sample of eight spindles reported here. The majority of human ECR spindles (75%) did not fall silent during movement to targets opposite to the preferred direction. In comparison, the majority of the models predict a period of zero firing rate. This difference between model predictions and human data could be improved by adding an EMG-linked gamma motor neuron activation term. This should be done in conjunction with human experiments that include a load at the wrist since the “non-forceful” contractions in these step-tracking data do not recruit the ECR muscle strongly. We hypothesize that by creating a load that would recruit the ECR muscle during this task, responses would switch from “hamstring-like” to “triceps surae-like.” The triceps surae muscles are more strongly recruited during locomotion and required an EMG-linked term to improve the prediction of the models (Prochazka and Gorassini, 1998b).

After convincing ourselves that the models had predictive value for human spindles, we simulated the random movement task. We had a number of objectives for pursuing these simulations. First, we wanted to address the issue of whether firing rates approaching those reported during cat locomotion would be predicted during human movements that were *ecologically valid*. The human experimental data has been criticized for being unnaturally slow, and this has been used as a possible explanation for the lower overall firing rates (Prochazka, 1999). Second, we wanted to use a task that could predict tuning properties of muscle spindles when joint position and velocity were dissociated. Finally, we wanted to use a task that generated data amenable to analysis by statistical methods other than the “mean vector” directional tuning approach. These objectives could have been achieved in a number of ways, but we chose to match the statistics of the movements to the ergonomics of typing.

### Importance of natural movement statistics?

What is the appropriate stimulus ensemble for evaluating the sensory information encoded in muscle spindle responses? If you subscribe to the notion that sensory neurons have adapted and evolved in an environment where some stimuli are more likely than others, then the answer is found in the statistics of that natural environment. Over the past decade, it has become clear that sensory encoding in the visual and auditory systems is specifically adapted to the statistics of natural environments (Simoncelli and Olshausen, 2001; Olshausen and Reinagel, 2003). In the primary visual system for example, it has been shown that models based on synthetic stimuli do not generalize to natural stimulus statistics (David et al., 2004). Based on these findings from other areas of sensory neurophysiology, we wanted to test the muscle spindle models with natural stimulus ensembles—in a statistical sense.

The random movement simulations resulted in a number of predictions that are amenable to testing. While we have ranked the models according to similarity of responses to human data in the center-out task, the firing rates predicted during random movements will allow more definitive ranking of the models against human data. The information-theoretic analysis was useful for estimating a lower boundary of information transmission by muscle spindles. Since the models output continuous functions, that are filters of the original spike train, the entropy in the firing rates is less than that of the original spike trains. Less entropy means a lower capacity for transmitting information about the stimulus.

### Conclusions

We have found that models based on cat muscle spindle primary afferents have some predictive value for predicting the dynamic and static features of human muscle spindle firing during a simple task. The remaining discrepancy between cat and human data is the lower overall mean firing rate in humans. These models were then used to predict the information content of natural wrist movements and the mutual information between kinematics and spindle firing. This analysis predicts that the information that is represented in spindle firing rate is more strongly weighted to velocity when compared to length. This prediction, which needs to be tested empirically, suggests that biomimetic neuroprosthetic systems should concentrate more on providing velocity feedback to central structures responsible for decoding muscle spindle signals.

### Conflict of interest statement

The authors declare that the research was conducted in the absence of any commercial or financial relationships that could be construed as a potential conflict of interest.
